# A Sufentanil-Based Rapid Cardiac Anesthesia Regimen in Children Undergoing Percutaneous Minimally-Invasive Intraoperative Device Closure of Ventricular Septal Defect

**DOI:** 10.21470/1678-9741-2019-0176

**Published:** 2020

**Authors:** Zeng-Chun Wang, Qiang Chen, Ling-Shan Yu, Liang-Wan Chen, Gui-Can Zhang

**Affiliations:** 1 Department of Cardiovascular Surgery, Union Hospital, Fujian Medical University, Fuzhou, Fujian, People’s Republic of China.

**Keywords:** Heart Septal Defects, Ventricular, Anesthesia, Cardiac Procedures, Echocardiography, Hemodynamics, Respiration, Artificial, Hospital Costs

## Abstract

**Objective:**

To assess the effectiveness and safety of fast-track cardiac anesthesia using the short-acting opioid sufentanil in children undergoing intraoperative device closure of ventricular septal defect (VSD).

**Methods:**

This retrospective clinical study included 65 children who underwent intraoperative device closure of VSD between January 2017 and June 2017. Patients were diagnosed with isolated perimembranous VSD by transthoracic echocardiography. Then, they were divided into two groups, group F (n=30), whose patients were given sufentanil-based fast-track cardiac anesthesia, and group C (n=35), whose patients were given conventional cardiac anesthesia. Perioperative clinical data were analyzed.

**Results:**

No significant differences were found between the preoperative clinical parameters and intraoperative hemodynamic indices between the two groups. In group C, compared with group F, the postoperative duration of mechanical ventilation, the length of stay in the intensive care unit, the length of hospital stay, and the hospital costs were significantly increased.

**Conclusion:**

In this retrospective study at a single center, sufentanil-based fast-track cardiac anesthesia was shown to be a safe and effective technique for minimally-invasive intraoperative device closure of VSD in children, which was performed with reduced in-hospital costs.

**Table:** 

Abbreviations, acronyms & symbols				
AV	= Atrioventricular		MAP	= Mean arterial pressure
CVP	= Central venous pressure		SD	= Standard deviation
ECG	= Electrocardiography		TEE	= Transesophageal echocardiography
HR	= Heart rate		TTE	= Transthoracic echocardiography
ICU	= Intensive care unit		VSD	= Ventricular septal defect

## INTRODUCTION

Ventricular septal defect (VSD) is a common type of congenital heart disease in children^[1]^. The standard surgical treatment for VSD includes surgical repair and percutaneous device closure. Recently, minimally-invasive intraoperative device closure of VSD has become widely used and it has several advantages that include reduced trauma, minimally-invasive percutaneous delivery, use of echocardiography guidance, ease of operation and medical training, reduced procedure time, rapid postoperative recovery, low medical costs, and ease of conversion to conventional surgical repair in the event of device failure^[2-5]^. Fast-track cardiac anesthesia has previously been shown to be effective for adult cardiac surgical patients, resulting in rapid postoperative recovery^[6,7]^.

Therefore, this retrospective study aimed to assess the effectiveness and safety of fast-track cardiac anesthesia using the short-acting opioid sufentanil in children undergoing intraoperative device closure of VSD.

## METHODS

### Clinical Evaluation and Patient Groups

This retrospective study analyzed the clinical data of 65 children who underwent minimally-invasive intraoperative device closure of VSD in the Department of Cardiovascular Surgery, Union Hospital, Fujian Medical University, from January 2017 to June 2017. All the patients were diagnosed with isolated perimembranous VSD without any other intracardiac structural abnormality by transthoracic echocardiography (TTE) and they had no extracardiac vascular malformations. They were divided into two groups: group F, whose patients underwent sufentanil-based fast-track cardiac anesthesia (n=30), and group C, whose patients underwent fentanyl-based conventional cardiac anesthesia (n=35). The choice of different anesthetic techniques was based on the experiences and decisions of the anesthesiologists and the operators. Perioperative clinical data of the patients in the two groups were analyzed and included the preoperative clinical parameters, intraoperative hemodynamic indices, postoperative duration of mechanical ventilation, length of stay in intensive care unit (ICU), length of hospital stay, perioperative complications, and in-patient hospital costs.

### Fast-Track Anesthesia (Group F)

Patients fasted before the procedure. Before entering the operating room, intramuscular ketamine (5 mg/kg) and midazolam (0.05 mg/kg) sedation were administered. In the operating room, all patients were monitored by electrocardiography (ECG) and the measurement of peripheral blood oxygen saturation. A peripheral venous route was established, and a 5% glucose infusion was given. The peripheral arterial puncture was performed to measure changes in arterial blood pressure. Induction of anesthesia included administering intravenous sufentanil (1 µg/kg) followed by cisatracurium besylate (0.5 mg/kg). After muscle relaxation was achieved, lidocaine gel was applied to the tip of the tracheal tube used for nasal intubation. Mechanical ventilation was maintained with pressure control. The end-tidal carbon dioxide partial pressure was monitored. Subclavian vein or internal jugular vein catheterization was established to measure the central venous pressure. The nasopharynx temperature was used to monitor the child’s body temperature and to ensure that it was maintained above 36.5ºC. Arterial blood gas was monitored to ensure the adequacy of ventilation. Anesthesia was maintained with sufentanil (1-2 µg/kg/h), using an intravenous pump, and sevoflurane (2%) inhalation, and both agents were adjusted according to the depth of anesthesia. Ropivacaine (0.375%) was used for local anesthesia during the sternal incision and after sternal closure.

### Conventional Anesthesia (Group C)

The preparation before anesthesia induction in group C was the same as that before fast-track anesthesia in group F. Anesthesia induction was performed with intravenous fentanyl (10 µg/kg) and cisatracurium besylate (0.5 mg/kg). Fentanyl (approximately 5 µg/kg/h) was used to maintain intraoperative anesthesia with an intravenous pump, and sevoflurane (2%) was administered by inhalation, with both agents adjusted according to the depth of anesthesia.

### Transesophageal Echocardiography and Intraoperative Device Closure of VSD

Following general anesthesia, the probe used for transesophageal echocardiography (TEE) was placed into the esophagus via mouth. A small 2-3 cm incision was made at the lower end of the sternum to access the thoracic cavity. The pericardium was opened to expose the free wall of the right ventricle. Intravenous heparin (1 mg/kg) was used for systemic heparinization. Under TEE guidance, the puncture site was selected and located, and purse-string sutures were placed around the puncture site. A guide wire was used to establish a delivery track across the right ventricle, the VSD, and the left ventricle. An occluder was delivered into the site of the VSD. The left and right-side discs of the occluder were deployed in turn to close the VSD. TEE was performed to confirm the position of the occluder. Neither a residual shunt nor aortic regurgitation was noted, and ECG showed no atrioventricular (AV) block, indicating that the procedure was performed successfully^[4]^. The patients were transferred to the ICU after the procedure. Tracheal extubation was performed in the ICU for all patients.

### Monitoring Parameters

Table 1 shows the preoperative clinical data of both study groups. Table 2 shows the hemodynamic parameters in the two groups, including heart rate, blood pressure, and central venous pressure before anesthesia induction, after intubation, during skin incision, during chest closure, and after extubation. Table 3 shows the duration of surgery, duration of mechanical ventilation, length of ICU stay, length of hospital stay, and hospital costs. Table 4 shows the incidence of postoperative complications. Following discharge from hospital, patients were followed up for a period of 9-12 months.

**Table 1:** Comparison of clinical data in both groups.

	Group F	Group C	*P*-value
Number of patients	30	35	
Males/females	16/14	18/17	0.774
Age (years)	3.3±1.5	4.0±2.1	0.789
Body weight (kg)	17.1± 4.1	16.7±5.4	0.491
VSD size (mm)	4.5±1.6	4.8±1.3	0.823
Occluder size (mm)	5.8±1.9	6.3±2.6	0.688

VSD=ventricular septal defect

**Table 2:** Comparison of interoperative hemodynamic data in both groups during the procedure.

		Group F	Group C	*P*-value
Anesthesia induction	CVP (mmH_2_O)	6.0±1.1	6.2±1.6	0.359
	MAP (mmHg)	90.8±10.4	96.4±14.7	0.731
	HR (beats/min)	120.4±19.5	119.6±17.5	0.623
After intubation	CVP (mmH_2_O)	5.8±0.8	5.9±1.9	0.891
	MAP (mmHg)	86.7±7.4	90.2±9.7	0.746
	HR (beats/min)	114.5±18.2	109.8±20.4	0.947
Skin incision	CVP (mmH_2_O)	6.2±1.2	6.3±2.2	0.898
	MAP (mmHg)	100.5±21.5	94.6±12.5	0.746
	HR (beats/min)	135.6±26.8	129.9±27.9	0.879
Chest closure	CVP (mmH_2_O)	6.1±1.5	6.0±1.8	0.965
	MAP (mmHg)	90.7±12.6	95.75±14.8	0.685
	HR (beats/min)	115.6±20.8	121.7±18.8	0.836
After extubation	CVP (mmH_2_O)	6.4±1.7	6.5±2.0	0.977
	MAP (mmHg)	97.5±10.2	92.2±11.9	0.769
	HR (beats/min)	113.7±22.5	119.9±17.5	0.801

CVP=central venous pressure; HR=heart rate; MAP=mean arterial pressure

**Table 3:** Comparison of interoperative and postoperative clinical data in both groups.

	Group F	Group C	*P*-value
Operative time (minutes)	38.3±10.5	40.1±13.0	0.531
Mechanical ventilation time (hours)	1.4±0.8	4.6±2.1	0.046
Intensive care time (hours)	6.0±3.5	13.1±8.7	0.038
Hospital stay (days)	2.3±0.7	4.7±1.8	0.047
Medical cost (10000RMB)*	2.7±0.8	3.9±1.9	0.049

* Costs in renminbi (the Chinese currency)

**Table 4:** Comparison of postoperative complications' clinical data in both groups.

	Group F	Group C	*P*-value
Death	0	0	
Complete atrioventricular block	0	0	
Occluder dislodgement	0	0	
Significant residual fistula	0	0	
Aortic valve regurgitation	0	0	
Hemolysis	0	0	
Thrombosis	0	0	
Pulmonary infection	1	7	0.041
Bronchospasm or laryngeal edema	0	5	0.031

### Statistical Analysis

Continuous data were described as mean ± standard deviation (SD). Comparison of the clinical parameters between the two study groups used the independent samples *t*-test. Nominal variables between the two groups were compared using Fisher's exact test. A *P*-value < 0.05 was assumed to be statistically significant.

### Ethical Approval and Consent to Participate

The present study was approved by the ethics committee of Fujian Medical University, People’s Republic of China, and adhered to the tenets of the Declaration of Helsinki. Additionally, written informed consent was obtained from the relatives of the patients.

### Availability of Data and Materials

Data sharing is not applicable to this article as no data sets were generated or analyzed during the current study.

## RESULTS

VSD was successfully closed in all 65 patients, and the overall immediate closure rate and closure at three months rate were 96.9% and 100%, respectively. Any immediate recurrence of the residual shunt was usually found as a small amount of flow through the device itself, which only required close medical observation. No patient in either group required conversion to surgical repair, experienced fatal AV block or aortic valve regurgitation, or required a blood transfusion. There was no cerebral complication, no occluder dislodgement, or other organ complications in the two groups. There were no anesthesia-related perioperative complications in the two study groups. During the follow-up period of 9-12 months, there were no delayed-onset complications of complete AV block or new occurrences of aortic valve regurgitation or cases of sudden death.

There were no significant statistical differences in the preoperative clinical parameters between the two groups (Table 1). Table 2 shows that the hemodynamic parameters were stable and were not statistically different between the two groups before induction, after intubation, during skin incision, during the opening of the thoracic cavity, during closure of the skin incision, and after extubation (*P*>0.05). There were no significant differences in the operation time between the two groups (Table 3). However, the postoperative duration of mechanical ventilation, the length of ICU stay, the length of hospital stay, and the hospital costs were significantly higher in the conventional cardiac anesthesia group (group C) when compared with the fast-track cardiac anesthesia group (group F) (*P*<0.05). Table 4 shows that there were no incidences of death, third-degree AV block, occluder dislodgement, aortic valve regurgitation, hemolysis, or thrombosis after the procedure in either group. However, fewer patients had postoperative pulmonary infections, bronchospasm, or laryngeal edema in the fast-track cardiac anesthesia group (group F) than in the conventional cardiac anesthesia group (group C).

## DISCUSSION

Large doses of opioids are used in conventional anesthesia to provide sufficient depth of anesthesia with stable hemodynamics. However, this regimen leads to postoperative respiratory depression and prolonged mechanical ventilation, which are associated with increased postoperative complications, prolonged hospital stay, and increased hospital costs. In 1993, Verrier et al.^[8]^ proposed the concept of fast-track anesthesia for cardiac surgery. Previously, Heard et al.^[9]^ had proposed removing the tracheal tube within six hours after pediatric cardiac surgery as a standard approach, using early extubation. Since then, the number of studies that have examined the feasibility and safety of fast-track anesthesia and the choice of anesthetic agents has gradually increased^[10-12]^. The fast-track anesthesia technique involves adjusting the use of anesthetic agents and modifying anesthetic techniques to achieve the appropriate depth of anesthesia, early recovery of postoperative spontaneous breathing, a shorter duration of mechanical ventilation, a shorter length of ICU stay, and a reduction in postoperative complications. Combining fast-track cardiac anesthesia with intraoperative device closure of VSD would be likely to benefit the rapid recovery of pediatric surgical patients.

The staff of the Department of Cardiovascular Surgery, Union Hospital, Fujian Medical University now have extensive clinical experience in intraoperative device closure of VSD^[4]^. As the findings of the present study have shown, the operative time was reduced, the clinical efficacy was good, and the postoperative recovery was rapid. The duration of surgery could be controlled to within 30-40 minutes. Effective and reasonable anesthesia reduced the side effects of anesthetic drugs, improved the efficacy of surgical treatment, and further shortened the length of hospital stay and treatment costs. The present study was performed to compare the effectiveness of the short-acting opioid sufentanil with the fentanyl, which is used in conventional cardiac surgery, to provide evidence for the selection of anesthetic agents for pediatric intraoperative device closure of VSD.

A study by Thomson et al.^[13]^ showed that sufentanil had a stronger analgesic effect than fentanyl (approximately 5-10 fold) and that its duration of action was approximately twice that of fentanyl. Sufentanil has been shown to be associated with more stable hemodynamics and is more suitable for anesthesia in cardiovascular surgery^[14,15]^. Also, when compared with fentanyl, sufentanil has a higher selectivity for binding to µ1 receptors^[14,15]^. Therefore, sufentanil is less likely to cause respiratory depression than fentanyl. The findings of the present study also demonstrated that in addition to maintaining hemodynamic stability during the procedure, children in the fast-track cardiac anesthesia group (group F) had a shorter duration of mechanical ventilation, shorter length of ICU stay, and fewer respiratory complications than those in the conventional anesthesia group (group C). Friesen et al.^[16]^ reported that children undergoing cardiac surgery under fast-track anesthesia had symptoms that included irritability and elevated blood pressure during extubation. The mechanism of these effects may be related to postoperative hyperalgesia caused by rapid tolerance of opioids. However, reasonable postoperative analgesia is necessary in children undergoing fast-track anesthesia and minimally-invasive intraoperative device closure of VSD. Therefore, in this study, ropivacaine (0.375%) was used for local anesthesia during the incision of the sternum and after sternal closure to provide sufficient postoperative analgesia^[17,18]^. Also, lidocaine gel was applied to the tip of the tracheal tube before intubation to reduce irritation of the airway, as previously described^[19,20]^. The findings of the present study showed no significant difference in hemodynamic changes during extubation between group F and group C, and the children did not complain of significant postoperative pain.

In conventional pediatric cardiac surgery, high-dose fentanyl is used for analgesia. However, clinical experience has shown that fentanyl can significantly increase postoperative respiratory depression and can result in excessive postoperative sedation^[21,22]^. The use of fentanyl results in prolonged postoperative mechanical ventilation and increased respiratory complications and it is not a suitable analgesic for fast-track cardiac anesthesia in pediatric cardiac surgery. The findings of the current study also showed that the duration of postoperative mechanical ventilation was higher than one hour in the conventional cardiac anesthesia group (group C) and that the length of ICU stay was significantly longer when compared with the fast-track cardiac anesthesia group (group F). Postoperative complications, including pulmonary infection, laryngeal edema, and bronchospasm were significantly increased in the group C compared with group F. However, no significant differences were observed between these two groups in terms of procedural complications, such as fatal AV block, new occurrence of aortic valve regurgitation, occluder dislodgement, and presence of a residual shunt. Although transient arrhythmias were reported in both groups during the procedure, cardiac rhythm returned to normal without sequelae. These results indicate that transient arrhythmias are not related to the choice of anesthetic technique.

The inhalational anesthetic used, sevoflurane, is a clear, colorless, aromatic liquid with properties that include a low blood:gas partition coefficient, fast elimination, and strong controllability^[23-25]^. Sevoflurane was used in the present study in combination with sufentanil to induce anesthesia. Previous studies have shown that volatile anesthetic agents have protective effects against myocardial ischemia-reperfusion injury and are safe for use in cardiovascular surgery. Also, sevoflurane does not cause an increase in airway secretions and meets the anesthesia requirements for short-term procedures, such as pediatric intraoperative device closure of VSD.

Mechanical ventilation can be beneficial for postoperative hemodynamics and homeostasis. However, the prolongation of mechanical ventilation is associated with increased complications that include dislodged tracheal tube, obstruction of airway secretions, laryngeal edema, and pulmonary infection, resulting in a reported mortality rate of up to 27%^[26]^. Meissner et al.^[27]^ reported that early extubation in patients undergoing fast-track anesthesia was safe and did not affect postoperative cardiac function. A study by Fischer et al.^[28]^ showed that the occurrence of ventilator-associated pneumonia after cardiac surgery in children was significantly correlated with the postoperative duration of intubation. Therefore, the use of the fast-track anesthesia technique in pediatric minimally-invasive intraoperative device closure of VSD has significance for early weaning from mechanical ventilation.

This study has several limitations. No standard dose of anesthetic agents was available for fast-track anesthesia because of the short history of use of this technique in pediatric minimally-invasive intraoperative device closure of VSD. In the cases selected for this study, device closure was predicted to be relatively easy to perform, according to preoperative and intraoperative echocardiography (TTE) and intraoperative TEE. The conventional cardiac anesthesia technique was used for patients for whom the device closure could be challenging. This is a retrospective and single-center study in which the grouping of patients was dependent on the preference of the surgeon and anesthesiologist. Therefore, a selection bias might have been present in this study. However, the results of this study might be used to guide clinical practice and should be supported by future prospective, multicenter, randomized studies to verify the findings of this preliminary retrospective study.

## CONCLUSION

In this retrospective study at a single center, sufentanil-based fast-track cardiac anesthesia was shown to be a safe and effective technique for minimally-invasive intraoperative device closure of VSD in children, and it was associated with significantly reduced respiratory complications, reduced duration of postoperative mechanical ventilation, reduced length of ICU stay and hospital stay, and reduced hospital costs.

**Table:** 

Author's roles & responsibilities	
ZCW	Substantial contributions to the conception or design of the work; or the acquisition, analysis, or interpretation of data for the work; drafting the work or revising it critically for important intellectual content; final approval of the version to be published
QC	Substantial contributions to the conception or design of the work; or the acquisition, analysis, or interpretation of data for the work; drafting the work or revising it critically for important intellectual content; final approval of the version to be published
LSY	Substantial contributions to the conception or design of the work; or the acquisition, analysis, or interpretation of data for the work; drafting the work or revising it critically for important intellectual content; final approval of the version to be published
LWC	Drafting the work or revising it critically for important intellectual content; final approval of the version to be published
GCZ	Drafting the work or revising it critically for important intellectual content; final approval of the version to be published
